# The Implementation of Behavior Change Techniques in mHealth Apps for Sleep: Systematic Review

**DOI:** 10.2196/33527

**Published:** 2022-04-04

**Authors:** Amber Carmen Arroyo, Matthew J Zawadzki

**Affiliations:** 1 Department of Psychological Sciences University of California Merced, CA United States

**Keywords:** behavior change techniques, sleep, mHealth, apps, digital health, mobile phone

## Abstract

**Background:**

Mobile health (mHealth) apps targeting health behaviors using behavior change techniques (BCTs) have been successful in promoting healthy behaviors; however, their efficacy with sleep is unclear. Some work has shown success in promoting sleep through mHealth, whereas there have been reports that sleep apps can be adverse and lead to unhealthy obsessions with achieving perfect sleep.

**Objective:**

This study aims to report and describe the use of BCTs in mHealth apps for sleep with the following research questions: How many BCTs are used on average in sleep apps, and does this relate to their effectiveness on sleep outcomes? Are there specific BCTs used more or less often in sleep apps, and does this relate to their effectiveness on sleep outcomes? Does the effect of mHealth app interventions on sleep change when distinguishing between dimension and measurement of sleep?

**Methods:**

We conducted a systematic review following PRISMA (Preferred Reporting Items for Systematic Reviews and Meta-Analyses) guidelines to review articles on mHealth app interventions for sleep published between 2010 and 2020.

**Results:**

A total of 12 studies met the eligibility criteria. Most studies reported positive sleep outcomes, and there were no negative effects reported. Sleep quality was the most common dimension of sleep targeted. Subjective measures of sleep were used across all apps, whereas objective measures were often assessed but rarely reported as part of results. The average number of BCTs used was 7.67 (SD 2.32; range 3-11) of 16. Of the 12 studies, the most commonly used BCTs were feedback and monitoring (n=11, 92%), shaping knowledge (n=11, 92%), goals and planning (n=10, 83%), and antecedents (n=10, 83%), whereas the least common were scheduled consequences (n=0, 0%), self-belief (n=0, 0%), and covert learning (n=0, 0%). Most apps used a similar set of BCTs that unfortunately did not allow us to distinguish which BCTs were present when studies reported more positive outcomes.

**Conclusions:**

Our study describes the peer-reviewed literature on sleep apps and provides a foundation for further examination and optimization of BCTs used in mHealth apps for sleep. We found strong evidence that mHealth apps are effective in improving sleep, and the potential reasons for the lack of adverse sleep outcome reporting are discussed. We found evidence that the type of BCTs used in mHealth apps for sleep differed from other health outcomes, although more research is needed to understand how BCTs can be implemented effectively to improve sleep using mHealth and the mechanisms of action through which they are effective (eg, self-efficacy, social norms, and attitudes).

## Introduction

### Background

Mobile health (mHealth) is the use of mobile technology (eg, smartphones) to improve health practices. mHealth interventions have incredible potential to implement large-scale health interventions at low cost, and their efficacy to promote health behaviors such as physical activity and diet has been established [1-4]. Despite the increasing awareness that sleep has a critical association with the development and progression of the largest killers in the United States (eg, heart disease, cancer, stroke, Alzheimer disease, and diabetes) [5,6] sleep has not been studied to the same extent as other health behaviors [7]. Notably, the use of mHealth apps for sleep across peer-reviewed studies has not been systematically reviewed.

There is a growing concern that apps should not be used for sleep because they can cause orthosomnia: an individual’s unhealthy obsession with achieving perfect sleep [8]. However, there are other reports that technology has the potential to improve sleep outcomes [9-11]. Possibly, the lack of consensus for the viability of mHealth apps with sleep is because of the components making up the intervention. The contents of all interventions are known as behavior change techniques (BCTs), which are designed to change or redirect the determinants that regulate behavior [12]. This study aims to examine the use of BCTs across mHealth apps for sleep to identify best practices for future mHealth app intervention development.

### BCTs in mHealth Apps for Sleep

#### Overview

BCTs are the irreducible active ingredients of all interventions used to facilitate behavior change [12]. The most common classification of BCTs is the *BCT Taxonomy V1* [12] and is widely considered the gold standard for behavior change research design and reporting [12-14]. The taxonomy defines 16 BCT clusters, with each representing a principal method of behavior change. Incorporating these evidence-based BCTs in interventions is recommended because they are known to successfully change health behaviors [15].

The efficacy of BCTs in mHealth apps for sleep has not been systematically examined despite some initial promise on their efficacy. One study did evaluate the number and type of BCTs used in commercially available sleep apps, reporting a greater number of BCTs used for physical activity compared with sleep, although sleep had a larger number of BCTs used than sedentary behavior [1]. However, they did not examine whether the number of BCTs was associated with better or worse treatment outcomes. The researchers also reported some of the BCTs used in mHealth apps for sleep were dissimilar to those seen in apps for physical activity (ie, social support and reward and threat), which may reflect a conscious or unconscious understanding by app developers that sleep is a unique construct. One particular feature that differentiates sleep from other health behaviors is that it is not always under an individual’s control. For example, an able-bodied individual can usually control how many steps they take in a day or how many calories they eat but cannot directly determine how many hours of sleep they get in a day or how many times they wake up in the middle of the night. As such, there may be some BCTs that are uniquely suited for mHealth apps for sleep. Although there is no overall framework for which BCTs might work best with sleep in mHealth apps, a brief overview is provided in the subsequent section to hypothesize possible associations.

#### Hypothesizing Differences in BCTs for Sleep

The implementation of BCTs that are aimed at changing aspects of behavior before sleep could be more appropriate for sleep than BCTs focused on future outcomes or consequences of sleep. This is because directing attention to the outcomes or consequences of sleep necessitates an anticipation of future events (ie, worry), which is often accompanied by anxiety. Anxiety is known to interfere with the successful initiation of sleep [16,17], as sleep often requires a quiet state of mind to be achieved and performed successfully [18]. Anxiety is also related to physiological arousal that disrupts the relaxation process needed for sleep [19-21]. Therefore, the BCTs that direct attention to the predictors of sleep may be optimal for sleep apps because they may bypass the arousal-related processes that could interfere with the initiation of sleep. BCTs focused on aspects of behavior before sleep are shaping knowledge, associations, repetition and substitution, antecedents, regulation, and self-belief. For example, antecedents can work to restructure or add objects to the physical environment to promote the behavior, such as creating a sleep sanctuary in one’s bedroom (eg, installing blackout curtains, white noise machine, or comfortable bedding). The BCT shaping knowledge can work to increase knowledge or skills to perform the behavior, such as providing information about antecedents that facilitate or harm sleep (eg, avoid caffeine within 6 hours of bed or avoid vigorous exercise within 2 hours of bed).

In opposition, the BCTs that direct an individual’s attention to the outcomes or consequences of sleep are natural consequences, comparison of outcomes, reward and threat, scheduled consequences, and covert learning. The use of these BCTs may be less appropriate for sleep. For example, natural consequences emphasizes the consequences of a behavior, such as highlighting the health consequences of inadequate sleep duration to discourage poor sleep practices, but could have the unintended effect of increasing anxiety surrounding sleep duration and inhibit one’s ability to relax enough to fall asleep. This may even be the case with positive consequences, such as the reward in the reward and threat BCT. For instance, if an individual is told they will receive a reward if they obtain 7 to 9 hours of sleep for an entire week, it may result in pressure or anxiety surrounding sleep and interfere with sleep onset or maintenance.

There are some BCTs that do not fit clearly into either category and therefore no hypotheses will be made for their frequency of use in mHealth apps for sleep. These BCTs include social support, comparison of behavior, goal setting, feedback and monitoring, and identity. More information on BCTs and examples of their implementation with sleep are provided in Multimedia Appendix 1.

#### Sleep Outcome Measures

When examining the use of BCTs in mHealth app interventions for sleep, it is important to consider how sleep is operationalized. It is possible that the way sleep outcomes have been measured in the previous research may partially explain why some sleep apps appear to be beneficial, whereas others appear to be harmful [22]. One common and well-supported approach to studying sleep health identifies five dimensions of sleep [23]: sleep quality (satisfaction with sleep), sleep duration (total amount of sleep acquired over a 24-hour period), sleep continuity (ease of falling asleep and staying asleep), sleep timing (placement of sleep in a 24-hour period), and sleepiness (ability to maintain wakefulness). Researchers have been advised to evaluate multiple dimensions of sleep concurrently to obtain a more accurate representation of an individual’s sleep health. For example, if an intervention increases sleep duration but sleep continuity decreases (ie, efficiency of time in bed in relation to time asleep), then the intervention may not be considered successful. Indeed, there are cases of interventions improving some dimensions of sleep whereas others remain the same (eg, sleep quality improves but sleep duration remains the same) [24]. The dimensions of sleep also have different associations with health [23], suggesting they are unique constructs and should be treated as such in reviews of the literature. It is therefore important to consider the dimension of sleep being targeted while examining mHealth app interventions for sleep.

In addition to sleep dimensions, the extent to which sleep health is subjectively or objectively measured may also be an important consideration. Subjective sleep is a self-reported appraisal of how one is sleeping and is assessed with retrospective questionnaires and sleep diaries [23]. Objective sleep is a measured observation of sleep parameters that is not directly controlled by the participant and is often assessed with remote behavioral and physiological technology [23]. There is a growing body of research suggesting that subjective and objective sleep measures may not be redundant. For example, multiple studies have reported that their subjective and objective measures of sleep continuity (specifically, the number of awakenings in a night) did not correlate with one another [25-27]. There have also been cases of individuals reporting subjectively defined insomnia but objectively normal sleep [28]. In addition, it has been noted that subjective and objective measures of sleep can differentially predict treatment efficacy in interventions for insomnia [29]. It is therefore recommended to use both subjective and objective methods of sleep measurement to obtain a comprehensive understanding of how an individual is sleeping [25,30]. This study will examine both sleep overall and also will distinguish between sleep dimension, and whether sleep outcomes were subjectively or objectively measured.

### This Study

The aim of this study is to report and describe the use of BCTs in mHealth app interventions for sleep in the peer-reviewed literature. Despite several systematic reviews examining mHealth interventions for chronic disease management and other health behaviors [2], there has not been a systematic review dedicated to examining BCTs in fully automated mHealth apps for sleep. One systematic review [9] evaluated the design engineering and implementation of mHealth apps for sleep disturbances but included papers with no quantitative evaluations of sleep (eg, apps that only measured and tracked sleep) and apps that required clinician input (ie, did not function autonomously). There have also been promising reviews on the efficacy of internet-delivered interventions for insomnia [31] but they notably did not review mobile apps that offer unique features including portability, touchscreen interactivity, and notifications and alerts [32].

Given the rapidly expanding public health issue of inadequate sleep [33] and the vast number of commercially available sleep apps compared with the small number of peer-reviewed studies of sleep apps [9], conducting a systematic review of fully automated mHealth apps for sleep in the peer-reviewed literature is required. In this systematic review, we aim to identify when and how BCTs were used in mHealth apps for sleep, with the hope of informing future app development and providing areas to focus on in future meta-analyses. We had three research questions (RQs):

1. How many BCTs are used on average in sleep apps, and does this relate to their effectiveness with sleep?
2. Are there specific BCTs used more or less often in sleep apps, and does this relate to their effectiveness with sleep?
3. Does the effect of mHealth app interventions on sleep change when distinguishing between dimension and measurement of sleep?

## Methods

### Article Inclusion Procedure

#### Search Strategy

The search strategy and study selection methods were adopted from prior research reviewing the effectiveness of mobile phone apps in achieving behavior change for a broad range of health behaviors [34]. A search of the PubMed database included all articles published between January 1, 2010, and January 1, 2020. The 2010 start date was specified to acknowledge that the creation of smartphone apps was relatively recent [35,36]. The PubMed database was chosen because of its strong usability for systematic reviews [36] and given the substantial portion of sleep studies occurring in medical research. The search string was specified as Title [sleep* OR insomnia*] AND Title/Abstract [smartphone OR phone OR mHealth OR eHealth OR telehealth OR mobile OR digital OR iPhone OR Android] AND Title/Abstract [CBT OR cognitive behavioral OR health behavior OR behavior change OR behavior modification OR health promotion OR health education OR preventative health care OR preventative medicine OR behavioral medicine OR behavioral health OR health-related behavior OR lifestyle change OR intervention OR medical informatics OR mHealth]. An asterisk next to a term denotes truncation (search all terms that have this root). Different spellings of behavior (eg, *behaviour*) were accounted for in the search settings.

#### Study Selection

The following inclusion criteria was used for study selection: (1) articles were sampled from an adult population (≥18 years) and published in English in a peer-reviewed journal. (2) Articles reported a comparison with the mHealth app for sleep (eg, within-person pretest vs posttest or between-group experimental group vs control group), and a null hypothesis test was conducted to see if there was a statistically significant difference between comparison groups. (3) Articles reported the effects of the mHealth app intervention on a measured sleep outcome. (4) The primary intervention tool was a fully automated mHealth app for sleep accessible from a smartphone. (5) The article reported using at least one BCT in its description of the mHealth app intervention for sleep.

### Article Coding Procedure

#### Behavior Change Techniques

All articles were independently coded by author ACA. The presence or absence of each BCT was coded to understand the frequency and use of BCTs across digital interventions for sleep (RQ1 and RQ2). The *BCT Taxonomy version 1* (BCTTv1) coding manual [12] and web-based training materials [37] were used to code for the presence or absence of BCTs. The coding manual contains labels, definitions, descriptions, and examples of each BCT category [12]. Before coding the articles, the coder ACA completed the web-based *BCTTv1* training through the official website [38]. As is common practice [39], a BCT was only coded if it was explicitly stated, if it was applied to the target behavior (ie, sleep improvement through various behaviors), and if its purpose in the mHealth app intervention was to change behavior (ie, not solely for data collection such as prompts or reminders to fill in a survey). If an article stated an external source should be retrieved for more information about components of their mHealth app for sleep, the source was followed and coded for BCTs relevant to the mHealth app used in the original study—these external sources often included preregistered protocols or electronic appendices with screenshots of the app. Frequency of a BCT’s use in an intervention was not coded per coding instructions from the *BCTTv1* starter pack manual [39] and also because tracking how often a user engages with features of an app would require a hands-on review study design.

#### Sleep Outcomes

##### Overview

Sleep outcomes were only coded if they were a target of the mHealth app intervention for sleep. For example, some articles included outcomes like sleep apnea to measure prevalence in the sample and generalize to a population but not as a target of the mHealth app intervention. In cases such as these, sleep apnea was not coded.

##### Sleep Outcome Effect Coding

To answer RQs 1-3, the total number of positive, negative, and null sleep outcomes reported for each intervention was coded. *Positive* referred to desirable or advantageous improvements in a sleep outcome, whereas *negative* referred to undesirable or harmful effects on sleep. For example, a significant decrease in wake after sleep onset, although inherently reported as a negative number, was coded as *positive* because reducing the cumulative time spent awake after initially falling asleep is an improvement for sleep health. *Null* was coded when an intervention had a nonsignificant effect on a sleep outcome (*P*>.05). Within-person change was assessed using baseline and postintervention scores of a sleep measure. If a study reported using 2 nights of objective data at baseline to account for *first night effects* [40], the second night was used as the baseline measure.

As an exploratory step to supplement interpretation of these effects, the size of the effect of the intervention on a sleep outcome was also coded as a Cohen's *d*. This statistic is recommended for use when an outcome variable is measured in different ways (ie, different scales used to measure sleep outcomes) [41]. The mean, SD, and sample size at baseline and posttest for each sleep outcome was used to calculate the effect size Cohen's *d* using standard formulas [42,43]. Although there were not enough articles to properly pool and test for moderators (as would be done in a meta-analysis), the addition of effect sizes was included to supplement understanding of the effect of digital interventions on sleep outcomes in this study. More on the extraction and calculation of effect size data is provided in Multimedia Appendix 2.

##### Sleep Outcome Operationalization Coding

To answer RQ3, 2 sets of codes were applied for all sleep outcomes. First, the dimensions of sleep—sleep quality, sleep duration, sleep continuity, sleep timing, and sleepiness—that the sleep outcome measure assessed. Second, the method of measurement—subjective or objective—that was used to capture the sleep outcome. A detailed overview of the reporting of sleep dimensions and methods of measurement across all studies is provided in Table 1.

**Table 1:** Dimensions of sleep, definitions, and measurement.

Sleep dimension	Definition [23]	Measurement	
		Subjective	Objective
Sleep quality	Satisfaction with sleep; subjective assessment of sleep as *good* or *poor*.	Insomnia Severity Index, Pittsburg Sleep Quality Index, Sleep Diary, Sleep Condition Indicator, and Jenkins Sleep Scale	—^a^
Sleep duration	The total amount of sleep acquired in a 24-hour period.	Sleep Diary	Actigraphy, WatchPat
Sleep continuity	The ease of falling asleep and returning to sleep (sleep efficiency, wake after sleep onset, sleep onset latency, and number of awakenings).	Sleep Diary	Actigraphy, WatchPat
Sleep timing	The placement or positioning of sleep in a 24-hour period.	Sleep Timing Questionnaire and Sleep Hygiene Index	—
Sleepiness or alertness	The facility to maintain or sustain attentive wakefulness.	Epworth Sleepiness Scale, Work Productivity and Impairment, Functional Outcomes of Sleep, Glasgow Sleep Impact Index	—

^a^Not available.

## Results

### Literature Search

**Figure 1:**
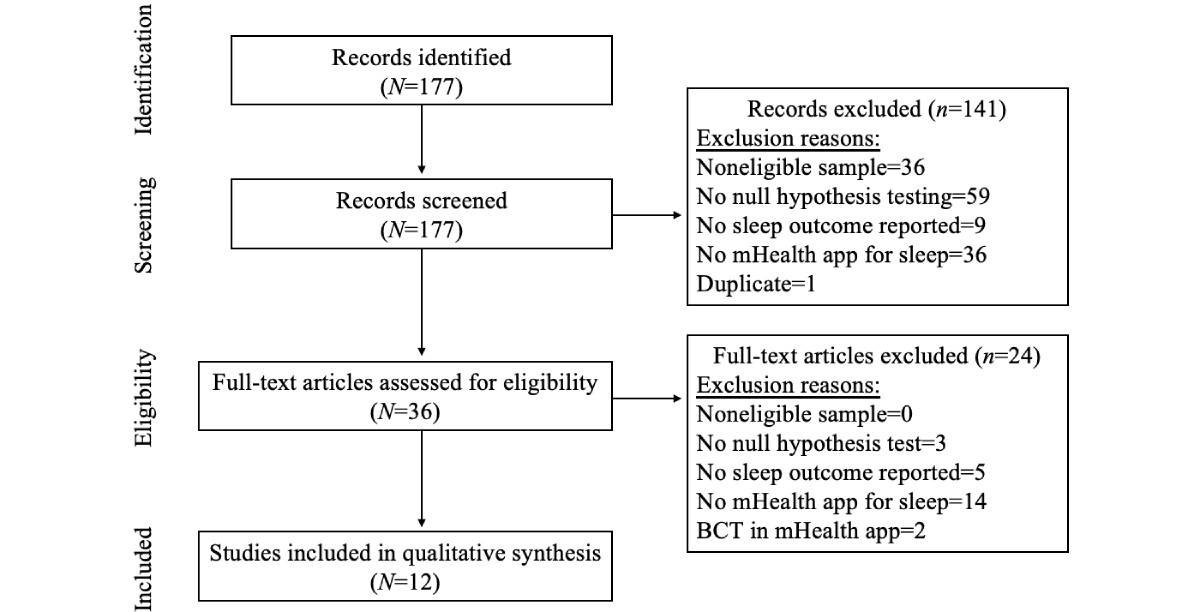
PRISMA (Preferred Reporting Items for Systematic Reviews and Meta-Analyses) reporting of article screening decisions. BCT: behavior change technique; mHealth: mobile health.

A total of 177 articles were identified from the PubMed database search conducted on January 9, 2020. Of the 177 articles, 141 (79.7%) were excluded in the title and abstract screening. Of the 36 articles that underwent full-text review, 24 (66.7%) were excluded, resulting in 12 eligible articles in this study. Figure 1 shows a detailed overview of article screening decisions in PRISMA (Preferred Reporting Items for Systematic Reviews and Meta-Analyses) format [44].

### Characteristics of Included Studies

All study characteristics are reported in Tables 2 and 3. Studies were distributed evenly among those using a randomized controlled trial (RCT) design (6/12, 50%) and those using a within-person pretest–posttest design (6/12, 50%). All studies (12/12, 100%) were conducted in developed countries: 42% (5/12) in the United States; 17% (2/12) each in the Netherlands and Australia; and 8% (1/12) each in Korea, England, and Taiwan. Most participants were middle aged (mean 39; range 22-48 years), 67% female (8/12, 67%, studies had a majority female sample), and all studies that reported ethnicity had a majority of White participants in their samples (although the majority of studies did not report ethnicity). The range of intervention duration was 2 to 26 weeks, although most interventions had an average duration of 6 to 7 weeks. Of the 12 studies, 8 (67%) included a validated measure of insomnia and 4 (33%) included measures of sleep quality or sleep condition in general. Of the 8 studies with insomnia measures, 6 (75%) had samples with clinically significant levels of insomnia and 2 (25%) had samples with subthreshold insomnia.

In preliminary analyses, we examined the effects of mHealth app interventions on sleep outcomes. Across 67 coded effects, 39 (58%) sleep outcomes were positive, 28 (42%) were null, and none (0%) were negative. Across the 12 studies, 4 (33%) reported only positive findings, 7 (58%) reported mixed findings (both positive and null), and 1 (8%) reported only null findings. There were noticeable differences in the reporting of positive sleep outcomes between RCTs and pretest–posttest designs. Specifically, 47% (18/38) of sleep outcomes reported in RCTs were positive compared with 72% (21/29) of sleep outcomes reported in pretest–posttests being positive. Of the studies reporting multiple effects, a majority of pretest–posttest studies reported only positive results (4/6, 67% pretest–posttest studies reported only positive effects with no null), whereas none (0/6, 0%) of the RCT studies reported only positive results. The majority (5/6, 83%) of RCTs reported both positive and null effects.

Next, we examined if baseline insomnia of participants played a role in the outcomes reported by interventions. Of the 8 samples with baseline measures of insomnia for their participants, 6 (75%) had clinically significant insomnia and 2 (25%) had subthreshold insomnia levels. No noticeable difference in the effect of mHealth app interventions on sleep between participants with clinical insomnia and subclinical insomnia was found. Specifically, of the 6 samples, 4 (67%) samples with clinical insomnia reported more positive sleep outcomes than null for their intervention, 1 (17%) reported an even ratio of positive to null sleep outcomes, and 1 (17%) reported more null compared with positive sleep outcomes. Of the 2 samples with subthreshold insomnia, 1 (50%) reported only positive sleep outcomes whereas the other (1/2, 50%) reported a nearly even number of positive and null sleep outcomes (6 positive and 7 null). Thus, there was not clear evidence that samples with varying levels of insomnia experienced fewer positive effects of the mHealth app interventions on their sleep.

**Table 2:** Characteristics of included studies (N=12; part 1).

Study	Design	Country^a^	Sample size^b^	Age (years), mean (SD)^b^	Female participants, n (%)^b^	Ethnicity of participants, n (%)^b^
Horsch et al [45]	RCT^c^	The Netherlands	151	40 (13)	94 (62)	Not reported
Pulantara et al [46]	Pre-post	United States	27	36 (10)	3 (11)	White: 20 (74)
Murawski et al [24]	RCT	Australia	160	42 (10)	128 (80)	White: 146 (91) and Asian: 10 (6)
Kang et al [47]	Pre-post	Korea	19	45 (10)	12 (63)	Not reported
Espie et al [48]	RCT	English-speaking	1711	48 (14)	1329 (78)	White: 1558 (91); mixed: 36 (2); other: 35 (2); Asian: 45 (3); and Black: 19 (1)
Reilly et al [49]	Pre-post	United States	38	44 (11)	6 (16)	White: 34 (90); Black: 2 (5); Puerto Rican: 2 (5); Filipino: 2 (5); American Indian: 1 (3); and other: 2 (5)
Luik et al [50]	Pre-post	England	98	44 (15)	65 (66)	Not reported
Oftedal et al [51]	RCT	Australia	40	36 (10)	21 (53)	White: 36 (90); Asian: 2 (5); Middle Eastern: 1 (2.5); and Aboriginal or Torres Straight or Pacific Islander: 1 (2.5)
van Drongelen et al [52]	RCT	The Netherlands	502	41 (8)	34 (7)	Not reported
Bostock et al [53]	RCT	United States	270	34 (6)	90 (33)	Not reported
Horsch et al [54]	Pre-post	The Netherlands	45	35 (14)	30 (67)	Not reported
Chu et al [55]	Pre-post	Taiwan	18	22 (1)	15 (83)	Not reported

^a^If country is not specified (ie, recruited via the web without a country requirement) then the language requirement is stated.

^b^Sample size and sample characteristics are at baseline (or posttest, if unavailable). Unaccounted percentages are nonresponders. Percentages over 100% represent biracial or multiple categories selected or attributed to rounding.

^c^RCT: randomized controlled trial.

**Table 3:** Characteristics of included studies (N=12; part 2).

Study	Duration in weeks	Baseline insomnia (ISI^a^ score)	Supplemental mode of delivery	Nonsleep intervention target	BCTs^b^ used in app
Horsch et al [45]	6-7	Insomnia (16.4)	None	None	1, 2, 3, 4, 7, 8, 9, 10, 11, 12, and 13
Pulantara et al [46]	4-6	Insomnia (15.6)	Human, in-person, phone call, SMS	None	1, 2, 3, 4, 7, 8, 9, and 11
Murawski et al [24]	12	No insomnia (12.4)	Email, mail, SMS	Physical activity	1, 2, 3, 4, 5, 7, 8, 9, and 12
Kang et al [47]	4	Insomnia (20.4)	Human, in-person, phone call	None	1, 2, 4, 6, 7, 8, 9, 11, and 12
Espie et al [48]	8	N/A^c^	Website	None	1, 2, 3, 4, 7, 8, 9, 11, and 12, 13
Reilly et al [49]	6	Insomnia (16.0)	App	None	1, 2, 4, 7, 8, 11, 12, and 13
Luik et al [50]	6 20-min sessions	Insomnia (18.5)	Human, phone call, website	None	1, 2, 3, 4, 7, 8, 9, 11, 12, and 13
Oftedal et al [51]	4	N/A	Email	Physical activity, diet	1, 2, 3, 4, 5, 9, and 12
van Drongelen et al [52]	26	N/A	Website	Physical activity, nutrition	4, 11, and 12
Bostock et al [53]	8	N/A	Website	None	1, 2, 3, and 9
Horsch et al [54]	3	No insomnia (13.5)	None	None	2, 4, 7, 8, 11, and 12
Chu et al [55]	2	Insomnia (18.5)	None	None	1, 2, 4, 7, 8, 11, and 12

^a^ISI: Insomnia Severity Index. Insomnia was defined using the ISI (≥15 indicates clinical insomnia) because of its reliability and validity to detect clinical cases of insomnia [56]. Scale range 0-28. Range 0-7, no clinically significant insomnia; range 8-14, subthreshold insomnia; range 15-21, clinical insomnia (moderate); and range 22-28, clinical insomnia (severe).

^b^BCT: behavior change technique. BCT numbers (column 5) are as follows: 1, goals and planning; 2, feedback and monitoring; 3, social support; 4, shaping knowledge; 5, natural consequences; 6, comparison of behavior; 7, associations; 8, repetition and substitution; 9, comparison of outcomes; 10, reward and threat; 11, regulation; 12, antecedents; 13, identity; 14, scheduled consequences; 15, self-belief; and 16, covert learning.

^c^N/A: not applicable. Studies that did not report a validated measure of insomnia for their treatment group.

Finally, when exploring effect sizes, the average sleep outcome effect size (Cohen's *d*) across digital interventions was 0.87 (range 0.04-2.88). However, of the 67 coded effects for sleep outcomes, only 39 (58%) had sufficient information to calculate a Cohen's *d* effect size (ie, mean, SD, and sample size at baseline and after the test). This severely limited our ability to provide an additional method of interpretation for the effect of digital interventions on sleep outcomes using effect sizes. Specifically, of the 12 studies, 2 (17%) were excluded because they did not report enough information to calculate an effect size for any of their sleep outcomes. Of the remaining 10 studies, we were still unable to calculate effect sizes for 42% of the reported sleep outcomes accounted for in the positive or null outcome coding. Therefore, this study uses the primary system of sleep outcome interpretation (positive or null outcome coding) for the *Results* section and provides further detail about the effect sizes in Multimedia Appendix 2.

### RQ1: The Number of BCTs Used in mHealth Apps for Sleep

RQ1 examined how many BCTs were typically used in studies and how participants slept in those studies. The average number of different BCTs used across interventions (N=12) was 7.67 of 16 (SD 2.32; range 11-3). Most interventions implemented several different BCTs, with 75% (9/12) of studies reporting using ≥7 BCTs in their mHealth app intervention for sleep. Increasing or decreasing the number of BCTs did not seem to produce a discernible pattern in the proportion of positive sleep outcomes (Table 4). As one way to compare, we grouped studies that used ≥8 or ≤7 BCTs, creating the split point at the average number of BCTs used in all studies. The average percentage of positive outcomes for studies using ≥8 BCTs was 66.7% positive (SD 26.56%) and the average for studies using ≤7 BCTs was 60% positive (SD 45.41%), indicating a negligible difference between the 2 groups. Moreover, both the groups (≥8 and ≤7 BCTs) had very high SDs, indicating a large within-group variability. For example, the 2 studies using 7 BCTs had low congruence, where 1 study had 0% positive outcomes and the other had 100% positive outcomes. Thus, this suggests that simply increasing or decreasing the total number of BCTs is not automatically related to more positive sleep outcomes.

**Table 4:** Number of behavior change techniques (BCTs) used in an intervention and sleep outcomes reported in those interventions (N=12).

Study	Number of BCTs	Sleep outcomes	
		Number of positive outcomes, n (%)^a^	Number of null outcomes, n (%)
Horsch et al [45]	11	5 (50)	5 (50)
Espie et al [48]	10	3 (60)	2 (40)
Luik et al [50]	10	1 (100)	0 (0)
Murawski et al [24]	9	6 (46)	7 (54)
Kang et al [47]	9	7 (78)	2 (22)
Pulantara et al [46]	8	4 (100)	0 (0)
Reilly et al [49]	8	3 (33)	6 (67)
Oftedal et al [51]	7	0 (0)	2 (100)
Chu et al [55]	7	5 (100)	0 (0)
Horsch et al [54]	6	1 (100)	0 (0)
Bostock et al [53]	4	3 (75)	1 (25)
van Drongelen et al [52]	3	1 (25)	3 (75)

^a^The percentage of positive outcomes for sleep was calculated using the number of outcomes that were positive divided by the total number of outcomes (sum of the number of positive results and number of null results) reported across studies using this number of BCTs.

### RQ2: The Type of BCTs Used in mHealth Apps for Sleep

RQ2 examined the use of specific BCTs across interventions and whether their presence was important for sleep outcomes. As reported in Table 5, the BCTs that appeared most often across mHealth app interventions for sleep were feedback and monitoring, and shaping knowledge. Other BCTs that were frequently implemented by most (≥75%) of the interventions were goals and planning, antecedents, associations, repetition and substitution, and regulation. Conversely, some BCTs were rarely or never used: natural consequences, comparison of behavior, reward and threat, scheduled consequences, self-belief, and covert learning.

As a consequence of the frequent use of the same BCTs across studies, we were unable to examine unique effects of BCTs on sleep outcomes. This collinearity among interventions using the same BCTs made it impossible to discern a pattern of positive or null outcomes associated with the use of specific BCTs in interventions. Instead, we examined if there was a presence effect: if the presence of specific BCTs in an intervention was associated with a greater percentage of positive sleep outcomes reported (Table 5). The BCT comparison of behavior stood out because when it was included in interventions the rate of positive sleep outcomes was higher (78%) than the rate of positive sleep outcomes for other BCTs (that seemed to center around 50%-60%). Another BCT that stood out was natural consequences because when it was included in studies, the rate of positive sleep outcomes was low (40%) compared with the others. However, it is important to note that the BCT comparison of behavior only had 1 study that used it and the BCT natural consequences only had 2 studies, and thus, we would need more evidence to know if this is a reliable effect of the BCT or random chance.

**Table 5:** Frequency of behavior change techniques (BCTs) used in mobile health app interventions across all studies (N=12).

BCT	Studies using BCT, n (%)	Positive outcomes, n (%)^a^	Total number of outcomes
Feedback and monitoring	11 (92)	38 (60)	63
Shaping knowledge	11 (92)	36 (57)	63
Goals and planning	10 (83)	37 (60)	62
Antecedents	10 (83)	32 (54)	59
Associations	9 (75)	35 (61)	57
Repetition and substitution	9 (75)	35 (61)	57
Regulation	9 (75)	30 (63)	48
Comparison of outcomes	8 (67)	29 (60)	48
Social support	7 (58)	22 (56)	39
Identity	4 (33)	12 (48)	25
Natural consequences	2 (17)	6 (40)	15
Comparison of behavior	1 (8)	7 (78)	9
Reward and threat	1 (8)	5 (50)	5
Scheduled consequences	0 (0)	—^b^	—
Self-belief	0 (0)	—	—
Covert learning	0 (0)	—	—

^a^The percentage of positive outcomes was calculated using the total number of outcomes that were positive divided by the total number of outcomes reported across all studies using this specific BCT.

^b^None of the studies used this BCT.

### RQ3: Results by Dimension and Measure of Sleep

To further our interpretation of how mHealth app interventions influence sleep, the effect that interventions had on sleep was further broken down by dimension. Sleep quality was by far the most frequently targeted dimension of sleep with all studies including a measure for this dimension. Sleep quality also had the most reliable improvement compared with other dimensions of sleep (n=24, 73% of the 33 sleep quality outcomes were positive). The remaining dimensions of sleep were infrequently targeted by interventions (less than half of the interventions reported them). The sleep dimension that was targeted least across studies was sleep timing (2/12, 17%, studies), followed by sleep duration (3/12, 25%, studies), sleep continuity (3/12, 25%, studies), and sleepiness (5/12, 42%, studies). These dimensions also had a lower rate of positivity compared with sleep quality. Specifically, 73% (24/33) of sleep quality outcomes were positive compared with 0% (0/3) of sleep duration, 50% (2/4) of sleep timing, 60% (6/10) of sleepiness, and 67% of sleep continuity. The disproportionate appearance of sleep quality (33/67, 49%, sleep outcomes) compared with the other sleep dimensions (range of 3-10 outcomes per dimension) render these differences speculative and more studies with greater representation of dimensions would be needed to make fair comparisons between dimensions.

Beyond dimension, we examined whether the effect of mHealth app interventions on sleep differed based on whether sleep was subjectively or objectively measured. All the 12 studies used at least one subjective measure of sleep. Surprisingly, although 42% (5/12) of studies mentioned using objective measures of sleep within their intervention, only 17% (2/12) of studies reported results for objectively measured sleep. This brings into question why some studies did not report all the sleep outcomes they collected. Here, we provide a description of how sleep was measured across studies, but we are unable to make meaningful interpretations because of limited reporting of objective sleep measures. The 2 studies that reported objective measures of sleep had an average sleep outcome positivity rate of 78% and 33%. These 2 studies were above and below the study-wide average positivity rate of 58% and therefore do not give a clear indication of whether mHealth app interventions’ ability to improve sleep outcomes differs when distinguishing between subjectively and objectively measured sleep. Multimedia Appendix 3 [24,45-55] details the subjective and objective measures used for sleep outcomes across studies.

## Discussion

### Principal Findings

The purpose of this review was to examine the use of mHealth apps for sleep published in the peer-reviewed literature. Specifically, we aimed to report and describe the use of BCTs in mHealth apps for sleep and if their effects on sleep outcomes varied by dimension and method of measurement. We found that studies most often reported positive sleep outcomes from their interventions, with no adverse effects reported. This finding suggested that mHealth apps for sleep can have desirable effects on sleep outcomes, and results were in line with previous research supporting the efficacy of mHealth apps to improve other health outcomes such as physical activity [57], sedentary behavior [58], and chronic disease management [59]. We also found that the most commonly measured dimension of sleep was sleep quality and that objective measures of sleep were vastly underrepresented compared with subjective measures of sleep.

Regardless of sleep dimension or measurement, there were no negative effects of interventions on sleep reported across all studies. This was surprising considering research on orthosomnia indicating some adverse effects of sleep apps [8,60]. Our results could mean that reports of orthosomnia may not be attributed to mHealth apps rather individual characteristics in a subset of the population (eg, people with severe insomnia not included in this review) or attributed to short-term effects of sleep apps that dissolve after a few uses (and therefore were not captured in the studies we reviewed). Alternatively, they could be because of measurement bias in which measures to capture adverse effects such as orthosomnia were not included in the studies we reviewed. Despite several reports of orthosomnia caused by mHealth app interventions [8,60], to our knowledge, scientists are yet to develop a measure capturing this adverse effect—meaning adverse effects could be happening but are not being captured. The lack of negative findings could also be because of bias in reporting attributed to selective reporting of sleep outcomes only showing positive effects. As is discussed in detail in the *Implications for Sleep Outcomes* section, there was extreme variability in the reporting of sleep scales and subscales across studies, which impeded our ability to fully assess subcomponents of RQ1 and RQ2.

RQ1 examined if there was a pattern in the number of BCTs used in mHealth app interventions for sleep. We found that most interventions used several different BCTs in their apps instead of focusing on only a few. This follows from the previous work on mHealth targeting other health outcomes demonstrating a wide variety of BCTs implemented [59,61]. However, we were unable to determine if using a wide variety of BCTs in sleep apps was advantageous for sleep outcomes. Having several BCTs in an intervention could increase the likelihood of at least one BCT being able to help an individual improve their sleep, or could also lead to a disjointed and unpredictable experience with the app and potentially result in adverse associations with sleep. A meta-analysis on eHealth interventions for alcohol consumption did suggest it is better to focus on specific, rather than several, BCTs [62], and this could hold true for sleep apps. However, whether there is an ideal number of BCTs to implement or if it varies between dimensions of sleep is unknown and should be a major focus of future research.

Beyond examining the number of BCTs, RQ2 examined whether there were specific BCTs used more or less often across all mHealth apps for sleep. Although there was overlap in the BCTs deployed across studies, there were some BCTs that noticeably appeared more often than others across interventions. The BCTs feedback and monitoring and goal setting are some of the most commonly implemented BCTs across mHealth app interventions targeting physical activity and diet [63], and this held in our review of mHealth apps for sleep. Furthermore, the BCTs that appeared in almost all the interventions included: shaping knowledge, antecedents, associations, repetition and substitution, and regulation. The widespread use of these BCTs supports the hypotheses that techniques focused on aspects of behavior before sleep (eg, education about antecedents, habit formation, reducing negative emotion) may be more suitable in mHealth for sleep than BCTs focusing on future outcomes or consequences of sleep.

Indeed, we found most of the BCTs omitted or rarely used tended to focus on outcomes or consequences of sleep, and included scheduled consequences, reward and threat, natural consequences, and covert learning. This could reflect an understanding that sleep differs from other health outcomes in that it is not always under one’s control and that it may be better for sleep apps to focus on predictors of sleep rather than the outcomes or consequences of it. The omission of comparison of behavior suggests that despite being a major component in other mHealth app interventions [64], social components may not be as relevant in mHealth for sleep because of its innately solitary nature. The omission of self-belief was initially surprising considering it does not focus on social components or outcomes of sleep. However, social cognitive theory [65] suggests that self-efficacy, similar to self-belief, works in tandem with outcome expectations (part of natural consequences), so it may not make sense to target one without the other [66]. It is therefore understandable why self-belief was not promoted because of its relationship with outcome expectancies (part of natural consequences). Although we were unable to assess if these BCTs were associated with worse outcomes, future research could conduct optimization trials [67] to understand which BCTs, or combinations of BCTs, are effective.

RQ2 also examined if the presence of specific BCTs was associated with a larger percentage of positive sleep outcomes reported. Although the conclusions we can draw are limited because of the small number of studies, there were 2 BCTs that stood out. The BCT comparison of behavior was used in studies with the highest proportion of positive results, whereas natural consequences was used in studies with the lowest proportion of positive results. Both findings would map on to hypotheses that focusing on aspects of behavior related to sleep may be more advantageous than focusing on consequences of sleep.

The first part of RQ3 examined the effect of mHealth app interventions by sleep dimension. Sleep quality was the most consistently targeted and improved dimension of sleep by mHealth apps. This finding is encouraging because previous research suggests improvements in sleep quality is the most important indicator of the restorative benefits of sleep [24,68]. This may partly explain why mHealth apps seem to be so good at improving sleep quality. Our results also bring into question why other dimensions of sleep such as sleep duration do not share the same reliable improvements as sleep quality, especially as sleep duration is the key indicator of sleep health used by medical professionals [69]. Our findings warrant future examination of whether all dimensions of sleep can be improved by mHealth apps or whether mHealth app efficacy varies by sleep dimension.

The second part of RQ3 examined whether the effect of mHealth app interventions was consistent across subjectively and objectively measured sleep outcomes. This was important to assess because of differences in the long-term health outcomes associated with subjective and objective sleep [70,71], and their potential to differentially predict treatment efficacy in insomnia intervention research [29]. Unfortunately, we were unable to answer this question because of limited information provided from articles (ie, less than half of the interventions using objective measures reported them in their results). A similar issue was noted in a recent systematic review of smartphone-delivered interventions for health behaviors [72]. This problem highlights an overarching issue with the reporting of sleep outcomes that made it difficult to assess and compare sleep outcomes across interventions.

### Implications for Sleep Outcomes

When testing our RQs, we came across issues related to sleep outcome reporting and interpretation. Many studies did not use thresholds that are significant based on clinical research and instead relied on statistical significance as an indication for whether a sleep outcome improved and the intervention *worked*. This is problematic because the statistical improvement of a sleep outcome does not equate to a clinically meaningful improvement. The former would not be considered effective if it were used by clinicians in a sleep practice. There are excellent thresholds for clinically meaningful change laid out by [22] (eg, cumulative time spent awake after initially falling asleep should be <30 minutes); however, they were not referenced in the interpretation of sleep data. Furthermore, there were sometimes misleading conclusions about sleep improvement because outcomes were assessed individually instead of in relation to other relevant outcomes. For example, a significant increase in total sleep time by itself appears desirable, but if sleep efficiency (total sleep time/time in bed) decreased or did not improve, this could be an adverse effect of the intervention as it could indicate individuals spent more time in bed restless.

There are models for how to use a more systematic and comprehensive approach to sleep measurement and reporting. For example, the work by Carney et al [73] was meant to facilitate comparison of sleep across studies by creating a standardized sleep diary, a popular measure in sleep research. Their work resulted in 9 items for the standard sleep diary that are used to calculate 8 critical sleep indices [73]. Despite this consensus in 2012, neither of the studies that used a sleep diary in this review reported all 8 indices. Unfortunately, this was not an isolated issue to sleep diaries, and selective reporting continued while examining other measures of sleep. There was extreme variability in reporting of scales’ global scores, subscales, and items both within and across studies using the same scales (Multimedia Appendix 3). For instance, 7 studies used the Pittsburg Sleep Quality Index, but 4 studies reported only the global score, 1 study reported the global and all subscales, 1 study reported the global and 1 of 7 subscales, and 1 study reported no global but just 3 of 7 subscales. Although most studies using the Insomnia Severity Index used the global score derived from its 7 items, 1 study derived a global score from just 5 of the 7 items and also reported 4 of the 5 collected items individually. This lack of consensus is problematic as it makes synthesizing findings across these studies while controlling for the type of outcome measure nearly impossible, thereby precluding approaches such as meta-analysis that are needed to inform medical decision-making.

The inconsistent reporting of sleep outcomes is concerning for many reasons, including that there was no explanation for why some metrics (subscales or individual items) were highlighted, whereas others were omitted. The selective reporting also brought into question issues related to reporting bias (eg, if the omitted sleep metrics did not support hypotheses). The finding that none of the RCTs reported only positive results whereas most pretest–posttest studies reported only positive results could support this point given most RCTs have preregistration, which deters selective reporting of outcomes. Although RCTs provide the highest level of evidence to make causal inferences [74], pretest–posttest designs have a benefit of requiring fewer resources to execute, although they also have drawbacks including their inability to control for third variables because of a lack of randomization. The difference in positive sleep outcome reporting by study design could suggest that pretest-posttest studies are inflating the effect of digital interventions on sleep and that RCTs present a more accurate and variable picture of the potential for mHealth app interventions to improve sleep. It could also be because of random chance—our sample of studies was relatively small and it is possible that a larger sample would neutralize this pattern and find no difference in reporting by study design. To answer this question, it is important to address selective reporting of sleep outcomes as a field, potentially through an increased requirement for preregistration, even for pretest-posttest study designs.

The selective reporting of sleep outcomes presents challenges for the field when trying to determine the effect sizes of mHealth app interventions on different domains of sleep across studies. Whether there were valid reasons for their omission was not explained, but regardless of reasoning the field would benefit from reporting all collected sleep metrics to increase transparency and examination across studies (ie, all possible metrics of recorded sleep data are reported somewhere like appendices). Sleep researchers could use work by Buysse [23], Buysse et al [75], and Morin [22] to create an ontology and taxonomy for consensus on sleep measurement and reporting. This would create a shared language and expedite communication of information and results so researchers are not advancing in silos but rather the field is advancing together with increased speed and efficiency. It is also of critical importance that these conversations include clinicians and researchers to promote the collaboration across domains through similarly defined variables of sleep and the importance designated to them.

### Limitations and Future Directions

Although this study had several strengths including being the first (to our knowledge) to systematically examine the use of BCTs in peer-reviewed studies of fully automated mHealth apps for sleep, there were notable limitations to our study. A limitation inherent to all systematic reviews is its equal treatment of studies regardless of sample and effect sizes. We tried to account for this by including the effect sizes of sleep outcomes when possible; however, limited data and overall consensus in reporting precluded a full understanding of the overall effect. A meta-analysis could provide a more complete picture because it weighs studies according to the effect and sample size and can detect heterogeneity in effect sizes to identify subgroups of people for whom the intervention is more or less effective (an approach that is critical to the increasingly employed precision medicine approach). In addition, we were unable to model the co-occurrence of BCTs used in interventions which could be hiding negative effects (eg, if one BCT is adverse and the other is beneficial, their effect would be null) or a potential synergistic effect where combinations of BCTs are more effective together than when used alone.

Similarly, it was not possible to test if co-occurring targets of the intervention (eg, promoting physical activity in conjunction with sleep) could moderate the efficacy of the intervention on sleep outcomes. It has been suggested that targeting multiple health behaviors together can lead to greater health improvements than targeting one alone [76] because of spillover effects (eg, transfer or gateway effects) in which success with one health behavior aids in the ability to succeed with a second health behavior [77]. This may be relevant for behaviors such as physical activity and sleep as they are known to have a reciprocal relationship with one another [78,79]. By contrast, targeting multiple health behaviors at once could fail to address either behavior in sufficient depth, thereby reducing the intervention’s potential to be effective [80-82]. This would be in line with theory that suggests addressing multiple health behaviors requires significant effort (cross-behavior regulation [83]) and that the effort put toward improving one health behavior could limit one’s ability to improve another (self-control strength model and ego depletion [84,85]).

Another potential confound we were unable to account for was the multi-model intervention delivery involving a human coach. Of the 12 studies, 3 (25%) had a live human component that may have enhanced overall effectiveness of the intervention on sleep outcomes. Indeed, there is some evidence to suggest that human guidance of internet-based interventions can improve intervention efficacy [86] and also promote engagement with the digital intervention [87]. Although there are benefits to adding human guidance, there are also potential drawbacks, including the addition of human support increasing cost of the intervention and burden on health care providers. Important factors to examine in regard to the addition of human support in digital interventions are whether the quantity of human support, quality of human support (ie, level of expertise), frequency, timing, or mode of delivery (in-person, via phone call, or video chat) matter for the overall efficacy of digital interventions on sleep.

Although it was not a focus of this study, an important next step is to examine the use of sleep hygiene in conjunction with BCTs. Sleep hygiene is a set of behavioral and environmental recommendations to promote sleep [88] and is often the first line of defense and treatment for sleep disorders [64,89,90]. Sleep hygiene is known to improve sleep in clinical and nonclinical populations [64], and it has been noted that popular commercial sleep apps are well-equipped to support sustainable sleep hygiene practices [10]. Improvements in sleep hygiene behaviors mediated sleep quality in a recent mHealth app intervention study [91] in which 30% of the changes in sleep quality were explained by changes in sleep hygiene. A natural extension and future direction of our study is adding a meta-analysis to examine efficacy while weighing according to sample sizes and identify the role of moderators of any observed effects (eg, sleep hygiene, co-occurring BCTs, or cotargeted health behaviors).

### Conclusions

This study conducted a systematic review of published peer-reviewed articles from 2010 to 2020 on mHealth app interventions for sleep, their use of BCTs, and their effect on sleep outcomes. Overall, we found overwhelming evidence that sleep apps can be effective at improving sleep, and we did not come across any reports of adverse effects (orthosomnia). However, this does not mean that adverse effects did not occur, and we recommend future research work on revising standards of sleep outcome measurement and reporting. We found evidence that the type of BCTs used in mHealth apps for sleep differed from other health outcomes, which suggests mHealth app intervention components may not be a one-size-fits-all and that sleep apps may require different design from other health app interventions. Further research is needed with improved measures and reporting of sleep to identify the optimal design components and potential limitations of mHealth app interventions for sleep.

## Appendix

Multimedia Appendix 1 Behavior change techniques and examples with sleep.

Multimedia Appendix 2 Cohen's *d* effect size interpretations.

Multimedia Appendix 3 Measures used for sleep outcomes across studies.

## Abbreviations

BCT: behavior change technique
BCTTv1: BCT Taxonomy version 1
mHealth: mobile health
PRISMA: Preferred Reporting Items for Systematic Reviews and Meta-Analyses
RCT: randomized controlled trial
RQ: research question
SD: standard deviation
