# Supplementary material for: The Implementation of Behavior Change Techniques in mHealth Apps for Sleep: Systematic Review
Source: JMIR Mhealth Uhealth. 2022 Apr 4;10(4):e33527. doi: 10.2196/33527 (PMC9132368; doi:10.2196/33527)
Supplement: Multimedia Appendix 1 [file mhealth_v10i4e33527_app1.docx]

**Appendix 1: Behavior Change Techniques and Examples with Sleep**

**Supplemental Table 1.** BCTs, implementation, and examples with sleep (*n* = 16).

| BCT and implementation | Example with sleep |
| --- | --- |
| (**1) Goals and Planning**  Goal setting (behavior), Problem solving, Goal setting (outcome), Action planning, Review behavior goal(s), Discrepancy between current behavior and goal, Review of outcome goal(s), Behavioral contract, Commitment | The app prompts the user to set a goal of getting at least 8 hours of sleep each night |
| **(2) Feedback and Monitoring**  Monitoring of behavior by others without feedback, Feedback on behavior, Self-monitoring of behavior, Self-monitoring of outcome(s) of behavior, Monitoring of outcome(s) of behavior without feedback, Biofeedback, Feedback on outcome(s) of behavior | The app displays a calendar with a star on the days a person slept at least 8 hours that night |
| **(3) Social Support**  Social support (unspecified), Social support (practical), Social support (emotional) | The app gives access to an online support group for people improving sleep health |
| **(4) Shaping Knowledge**  Instruction on how to perform a behavior, Information about antecedents, Reattribution, Behavioral experiments | The app provides instructions on how to create a consistent sleep schedule to help promote sleep health |
| **(5) Natural Consequences**  Information about health consequences, Salience of consequences, Information about social and environmental consequences, Monitoring of emotional consequences, Anticipated regret, Information about emotional consequences | The app explains that not getting enough sleep is associated with increased morbidity and mortality |
| **(6) Comparison of Behavior**  Demonstration of the behavior, Social comparison, Information about others’ approval | The app displays the number of nights other users get 8 hours of sleep and compares it to the user’s sleep data |
| **(7) Associations**  Prompts/cues, Cue signaling reward, Reduce prompts/cues, Remove access to reward, Remove aversive stimulus, Satiation, Exposure, Associative learning | The app sends a push notification with a unique sound alert to cue the user that it is time to get ready for bed |
| **(8) Repetition and Substitution**  Behavioral practice/rehearsal, Behavior substitution, Habit formation, Habit reversal, Overcorrection, Generalization of target behavior, Graded tasks | The app asks the user to increase their average total sleep time by 15 minutes the first week, and then increases by 15 minutes each week until they have reached an average 8 hours of sleep |
| **(9) Comparison of Outcomes**  Credible source, Pros and cons, Comparative imagining of future outcomes | The app advises the user to list and compare the advantages and disadvantages of sleeping a full 8 hours each night |
| **(10) Reward and Threat**  Material incentive (behavior), Material reward (behavior), Non-specific reward, Social reward, Social incentive, Non-specific incentive, Self-incentive, Incentive (outcome), Self-reward, Reward (outcome), Future punishment | The app’s virtual sleep coach congratulates the user for each day they get 8 hours of sleep |
| **(11) Regulation**  Pharmacological support, Reduce negative emotions, Conserving mental resources, Paradoxical instructions | The app advises the user on the use of stress management exercises to facilitate sleep onset |
| **(12) Antecedents**  Restructuring the physical environment, Restructuring the social environment, Avoidance/reducing exposure to cues for the behavior, Distraction, Adding objects to the environment, Body changes | The app instructs the user to install blackout curtains in their bedroom so external lights do not disrupt their sleep |
| **(13) Identity**  Identification of self as role model, Framing/reframing, Incompatible beliefs, Valued self-identity, Identity associated with changed behavior | The app suggests the user think of the tasks as reducing daytime fatigue rather than increasing their total sleep time |
| **(14)** **Scheduled Consequences**  Behavior cost, Punishment, Remove reward, Reward approximation, Rewarding completion, Situation-specific reward, Reward incompatible behavior, Reward alternative behavior, Reduce reward frequency, Remove punishment | The app arranges for money to be withdrawn from their bank account when the user does not achieve their goal of 8hrs of sleep |
| **(15) Self-Belief**  Verbal persuasion about capability, Mental rehearsal of successful performance, Focus on past success, Self-talk | The app advises the user to imagine themselves falling asleep simply and peacefully |
| **(16) Covert Learning**  Imaginary punishment, Imaginary reward, Vicarious consequences | The app advises the user to imagine the consequences of not sleeping well like being extremely tired the next day |
