# Supplementary material for: The Implementation of Behavior Change Techniques in mHealth Apps for Sleep: Systematic Review
Source: JMIR Mhealth Uhealth. 2022 Apr 4;10(4):e33527. doi: 10.2196/33527 (PMC9132368; doi:10.2196/33527)
Supplement: Multimedia Appendix 2 [file mhealth_v10i4e33527_app2.docx]

**Appendix 2: Cohen’s *d* Effect Size Interpretations**

**Method**

***Sleep Outcome Effect Size Extraction and Calculation***

During coding it was noticed that among the articles that reported effect sizes, the Cohen’s *d* effect sizes calculated from article-reported means, standard deviations, and sample sizes did not always match the in-text reported effect sizes or Cohen’s *d* from the authors. In an effort to use the same formula and be consistent across studies, we did not extract author-reported effect sizes. Instead, we calculated effect sizes for studies reporting the mean, standard deviation, and sample size for sleep outcome measures at baseline and posttest.

**Results**

***Research Question 1: The Number of BCTs used in mHealth Apps for Sleep***

Examining RQ1 with effect sizes (Supplemental Table 2) with the same grouping as we did for positive/null outcome system, the average effect size for studies using ≥8 BCTs was considered large at 1.07 while the average effect size for studies using ≤7 BCTs was considered medium at 0.40. However, it is important to note that two of the five studies that were originally included in the ≤7 BCTs calculations could not be included in the average effect size calculations, resulting in a unbalanced comparison (seven studies with ≥8 BCTs versus three studies with ≤7 BCTs). This limitation is compounded with that fact that effect sizes were calculated with 42% fewer sleep outcomes than the positive/null coding system. There was a similar pattern of within-group variability for effect sizes as seen with the positive/null outcome coding system. For example, of the two studies that used 10 BCTs in their interventions, one had an average effect size of 0.49 while the other had a much larger effect size at 2.86.

**Supplemental Table 2.** Number of BCTs used in an intervention and sleep outcomes reported in those interventions (*n* = 12 studies).

|  | 11  BCTs  (*n*=1) | | 10  BCTs  (*n*=2) | |  | | 9  BCTs  (*n*=2) | |  | 8  BCTs  (*n*=2) | | |  | 7  BCTs  (*n*=2) | |  | 6 BCTs  (*n*=1) | | 4 BCTs  (*n*=1) | | 3 BCTs  (*n*=1) | |
| --- | --- | --- | --- | --- | --- | --- | --- | --- | --- | --- | --- | --- | --- | --- | --- | --- | --- | --- | --- | --- | --- | --- |
| **Cohen’s *d*** | | 0.56 | 0.49 | 2.86 | | 0.36 | | 1.27 | | | 1.35 | 0.58 | | NA | 0.81 | | | 0.35 | | NA | | 0.04 |

*Note:* Cohen’s *d* values are derived from the mean absolute value of Cohen’s *d* effect sizes for all sleep outcomes used in studies using that particular number of BCTs. “NA” indicates there was insufficient information to calculate an effect size for any sleep outcomes reported in this study.

***Research Question 2: The Type of BCTs used in mHealth Apps for Sleep***

Examining BCTs by effect size (Supplemental Table 3), all BCT effect sizes are considered large (*d* ≥ 0.80) with the exception of “Natural consequences” and “Reward and threat” which would both be considered medium effect sizes. While these findings somewhat follow the pattern of results from the positive/null coding system, it shares the same limitations in terms of sample size (i.e., only one study each used each of the BCTs), which is compounded with the fact that only 39 sleep outcomes were used to compute effect sizes compared to 67 sleep outcomes in the positive/null coding system (i.e., 42% fewer sleep outcomes, 10 studies instead of 12 due to insufficient reporting for effect size calculations).

**Supplemental Table 3.** Frequency of BCTs used in mHealth apps across studies (*n* = 12).

| BCT | Average effect size  Cohen’s *d* (*n*) |
| --- | --- |
| Feedback & Monitoring | 1.03 (*n* = 9) |
| Shaping Knowledge | 0.87 (*n* = 10) |
| Goals & Planning | 1.03 (*n* = 8) |
| Antecedents | 0.81 (*n* = 9) |
| Associations | 0.96 (*n* = 9) |
| Repetition & Substitution | 0.96 (*n* = 9) |
| Regulation | 0.92 (*n* = 9) |
| Comparison of Outcomes | 1.14 (*n* = 6) |
| Social Support | 1.12 (*n* = 5) |
| Identity | 1.12 (*n* = 4) |
| Natural Consequences | 0.36 (*n* = 1) |
| Comparison of Behavior | 1.27 (*n* = 1) |
| Reward & Threat | 0.56 (*n* = 1) |
| Scheduled consequences | -- |
| Self-Belief | -- |
| Covert Learning | -- |

Note: *n* represents the number of studies (out of 12) using this specific BCT in their intervention. The average effect size for each BCT was calculated by taking the mean of effect sizes for all outcomes reported by interventions using that specific BCT.
