# Supplementary material for: The Implementation of Behavior Change Techniques in mHealth Apps for Sleep: Systematic Review
Source: JMIR Mhealth Uhealth. 2022 Apr 4;10(4):e33527. doi: 10.2196/33527 (PMC9132368; doi:10.2196/33527)
Supplement: Multimedia Appendix 3 [file mhealth_v10i4e33527_app3.docx]

**Appendix 3: Measures used for Sleep Outcomes Across Studies**

**Supplemental Table 4.** Subjective measures used for sleep outcomes across studies.

|  | |  | |  | | |  | |  | |  | Study ID^a^ | | | | | | | | |  | | |  |  | | |  |  | | |  | |
| --- | --- | --- | --- | --- | --- | --- | --- | --- | --- | --- | --- | --- | --- | --- | --- | --- | --- | --- | --- | --- | --- | --- | --- | --- | --- | --- | --- | --- | --- | --- | --- | --- | --- |
| Subjective measure | | 1 | | 2 | | | 3 | | 4 | | 5 | | | 6 | | 7 | | 8 | | 9 | | | 10 | | | | 11 | | | | 12 | | |
| **Insomnia Severity Index** | | | | | | | | | | | | | | | | | | | | | | | | | | | | | | | | | |
| Global score | + | | + | | | + | | + | |  | | | + | | + | |  | |  | | |  | | | | + | | | |  | | |  |
| 1: Falling asleep |  | |  | | |  | |  | |  | | |  | |  | |  | |  | | |  | | | |  | | | | + | | |  |
| 2: Staying asleep |  | |  | | |  | |  | |  | | |  | |  | |  | |  | | |  | | | |  | | | |  | | |  |
| 3: Waking up too early |  | |  | | |  | |  | |  | | |  | |  | |  | |  | | |  | | | |  | | | |  | | |  |
| 4: Satisfied |  | |  | | |  | |  | |  | | |  | |  | |  | |  | | |  | | | |  | | | |  | | |  |
| 5: Interfere |  | |  | | |  | |  | |  | | |  | |  | |  | |  | | |  | | | |  | | | | + | | |  |
| 6: Noticeable |  | |  | | |  | |  | |  | | |  | |  | |  | |  | | |  | | | |  | | | | + | | |  |
| 7: Worried |  | |  | | |  | |  | |  | | |  | |  | |  | |  | | |  | | | |  | | | | + | | |  |
| *5/7 Items for global,*  *however 4/5 reported* |  | |  | | |  | |  | |  | | |  | |  | |  | |  | | |  | | | |  | | | | + | | |  |
| **Pittsburg Sleep Quality Index** | | | | | | | | | | | | | | | | | | | | | | | | | | | | | | | | | |
| Global score | + | | + | | | + | | + | |  | | | + | |  | | null | |  | | |  | | | |  | | | |  | | |  |
| 1: Sleep quality |  | |  | | | + | |  | |  | | |  | |  | |  | |  | | |  | | | |  | | | |  | | |  |
| 2: Sleep latency |  | |  | | | + | |  | |  | | |  | |  | |  | | null | | |  | | | |  | | | |  | | |  |
| 3: Sleep duration |  | |  | | | null | |  | |  | | |  | |  | |  | | null | | |  | | | |  | | | |  | | |  |
| 4: Sleep efficacy |  | |  | | | null | | + | |  | | |  | |  | |  | |  | | |  | | | |  | | | |  | | |  |
| 5: Sleep disturbance |  | |  | | | null | |  | |  | | |  | |  | |  | |  | | |  | | | |  | | | |  | | |  |
| 6: Use of sleep  medication |  | |  | | | null | |  | |  | | |  | |  | |  | | null | | |  | | | |  | | | |  | | |  |
| 7: Daytime dysfunction |  | |  | | | null | |  | |  | | |  | |  | |  | |  | | |  | | | |  | | | |  | | |  |
| *Addendum for PTSD* |  | | + | | |  | |  | |  | | |  | |  | |  | |  | | |  | | | |  | | | |  | | |  |
| **Sleep Diary** | | |  | |  |  | |  | |  | | |  | |  | |  | |  | | |  | | | |  | | | |  | | |  |
| Time in bed | | null | |  |  | | null | |  | | |  | |  | |  | |  | | |  | | | |  | | | |  | | |  |  |
| Total sleep time | | null | |  |  | | null | |  | | |  | |  | |  | |  | | |  | | | |  | | | |  | | |  |  |
| Wake after sleep onset | | + | |  |  | |  | |  | | |  | |  | |  | |  | | |  | | | |  | | | |  | | |  |  |
| Sleep onset latency | | null | |  |  | | + | |  | | |  | |  | |  | |  | | |  | | | |  | | | |  | | |  |  |
| Sleep efficiency | | + | |  |  | | + | |  | | |  | |  | |  | |  | | |  | | | |  | | | |  | | |  |  |
| Sleep quality | |  | |  |  | |  | |  | | |  | |  | |  | |  | | |  | | | |  | | | |  | | |  |  |
| Terminal wakefulness | | null | |  |  | |  | |  | | |  | |  | |  | |  | | |  | | | |  | | | |  | | |  |  |
| Total wake time | |  | |  |  | |  | |  | | |  | |  | |  | |  | | |  | | | |  | | | |  | | |  |  |
| *Item #4- How many times did you wake up?* | | + | |  |  | |  | |  | | |  | |  | |  | |  | | |  | | | |  | | | |  | | |  |  |
|  | | |  | |  |  | |  | |  | | |  | |  | |  | |  | | |  | | | |  | | | |  | | |  |

^a^ 1=Horsch et al [45]; 2=Pulantara et al [46]; 3=Murawski et al [24]; 4=Kang et al [47]; 5=Espie et al [48]; 6=Reilly et al [49]; 7=Luik et al [50]; 8=Oftedal et al [51]; 9=van Drongelen et al [52]; 10=Bostock et al [53]; 11=Horsch et al [54]; 12=Chu et al. [55].

|  |  |  |  |  |  | | Study ID^a^ | | | | | |  | |  |  | |  |  | |  |
| --- | --- | --- | --- | --- | --- | --- | --- | --- | --- | --- | --- | --- | --- | --- | --- | --- | --- | --- | --- | --- | --- |
| Subjective measure | 1 | 2 | 3 | | | 4 | | 5 | 6 | 7 | 8 | 9 | | 10 | | | 11 | | | 12 | |
| **Sleep Condition Indicator** |  |  |  | | |  | |  |  |  |  |  | |  | | |  | | |  | |
| Global score |  |  |  | | |  | | + |  |  |  |  | | + | | |  | | |  | |
| **Sleep Hygiene Index** |  |  |  | | |  | |  |  |  |  |  | |  | | |  | | |  | |
| Global score |  |  | + | | |  | |  |  |  | null |  | |  | | |  | | |  | |
| **Epworth Sleepiness Scale** |  |  |  | | |  | |  |  |  |  |  | |  | | |  | | |  | |
| Global score |  | + | null | | |  | | null |  |  |  |  | |  | | |  | | |  | |
| *“A single item derived*  *from ESS about*  *falling asleep without*  *intending to.”* |  |  |  | | |  | |  |  |  |  |  | | + | | |  | | |  | |
| **Work Productivity and Impairment** | | | | | | | | | | | | | | | | | | | | | |
| Presenteeism |  |  |  | | |  | | + |  |  |  |  | | + | | |  | | |  | |
| Absenteeism |  |  |  | | |  | | null |  |  |  |  | | null | | |  | | |  | |
| **Dysfunctional Beliefs about Sleep** | | | | | | | | | | | | | | | | | | | | | |
| Global score | null |  |  | | | + | |  |  |  |  |  | |  | | |  | | |  | |
| **Sleep Timing Questionnaire** | | | | | | | | | | | | | | | | | | | | | |
| Waketime |  |  | + | | |  | |  |  |  |  |  | |  | | |  | | |  | |
| Bedtime |  |  | null | | |  | |  |  |  |  |  | |  | | |  | | |  | |
| **Functional Outcomes of Sleep-10** | | | | | | | | | | | | | | | | | | | | | |
| Global score |  |  |  | | |  | |  | + |  |  |  | |  | | |  | | |  | |
| **Jenkins Sleep Scale** |  |  |  | | |  | |  |  |  |  |  | |  | | |  | | |  | |
| Global score |  |  |  | | |  | |  |  |  |  | + | |  | | |  | | |  | |
| **Glasgow Sleep Impact Index** | | | | | | | | | | | | | | | | | | | | | |
| Global score |  |  |  | | |  | | + |  |  |  |  | |  | | |  | | |  | |

^a^ 1=Horsch et al [45]; 2=Pulantara et al [46]; 3=Murawski et al [24]; 4=Kang et al [47]; 5=Espie et al [48]; 6=Reilly et al [49]; 7=Luik et al [50]; 8=Oftedal et al [51]; 9=van Drongelen et al [52]; 10=Bostock et al [53]; 11=Horsch et al [54]; 12=Chu et al. [55].

**Supplemental Table 5.** Objective measures used for sleep outcomes across studies.

|  | | |  |  |  |  |  | Study ID^a^ | | | |  |  |  |  |  |  |
| --- | --- | --- | --- | --- | --- | --- | --- | --- | --- | --- | --- | --- | --- | --- | --- | --- | --- |
| Objective measure | | | 1 | 2 | 3 | 4 | 5 | 6 | 7 | 8 | 9 | | | 10 | | 11 | 12 |
| **Actigraphy** | | |  |  |  |  |  |  |  |  |  | | |  | |  |  |
| Sleep efficiency | |  |  |  | + |  |  |  |  |  | | |  | |  |  |  |
| Total sleep time | |  |  |  |  |  |  |  |  |  | | |  | |  |  |  |
| Wake after sleep onset | |  |  |  |  |  |  |  |  |  | | |  | |  |  |  |
| Time in bed | |  |  |  |  |  |  |  |  |  | | |  | |  |  |  |
| Sleep onset latency | |  |  |  |  |  |  |  |  |  | | |  | |  |  |  |
| #Awakenings/Restlessness | |  |  |  |  |  |  |  |  |  | | |  | |  |  |  |
| **WatchPat** | | |  |  |  |  |  |  |  |  |  | | |  | |  |  |
| % light & total mins |  |  |  |  |  | null |  |  |  | | |  | |  |  |  |  |
| % deep & total mins |  |  |  |  |  | null |  |  |  | | |  | |  |  |  |  |
| % REM & total mins |  |  |  |  |  | null |  |  |  | | |  | |  |  |  |  |
| Sleep efficiency |  |  |  |  |  | null |  |  |  | | |  | |  |  |  |  |
| Total sleep time |  |  |  |  |  | null |  |  |  | | |  | |  |  |  |  |
| Numb. of awakenings |  |  |  |  |  | null |  |  |  | | |  | |  |  |  |  |
| Sleep onset latency |  |  |  |  |  |  |  |  |  | | |  | |  |  |  |  |
| Time in bed |  |  |  |  |  |  |  |  |  | | |  | |  |  |  |  |

^a^ 1=Horsch et al [45]; 2=Pulantara et al [46]; 3=Murawski et al [24]; 4=Kang et al [47]; 5=Espie et al [48]; 6=Reilly et al [49]; 7=Luik et al [50]; 8=Oftedal et al [51]; 9=van Drongelen et al [52]; 10=Bostock et al [53]; 11=Horsch et al [54]; 12=Chu et al. [55].
